# A new species of *Khorata* Huber, 2005 (Araneae, Pholcidae) from Jiangxi Qiyunshan National Nature Reserve, southern China

**DOI:** 10.3897/BDJ.12.e141018

**Published:** 2024-11-19

**Authors:** Guchun Zhou, Jian Lu, Yutong Deng

**Affiliations:** 1 School of Life Sciences, National Navel Orange Engineering Research Center, Gannan Normal University, Ganzhou, China School of Life Sciences, National Navel Orange Engineering Research Center, Gannan Normal University Ganzhou China; 2 Administration of Jiangxi Qiyunshan National Nature Reserve in Jiangxi, Ganzhou, China Administration of Jiangxi Qiyunshan National Nature Reserve in Jiangxi Ganzhou China

**Keywords:** Asia, biodiversity, daddy-long-leg spiders, morphology, taxonomy

## Abstract

**Background:**

*Khorata* Huber, 2005 contains 52 species distributed in Cambodia, China, Laos, Thailand and Vietnam, out of which about 34 species have been recorded from China. It can be distinguished from all other genera of Pholcidae by the male chelicerae with lateral ledges and the cheliceral apophyses with cuticular ridges or scales. The femur of the male palp features a retrolateral apophysis. The male palp is small, characterised by a prolaterally attached genital bulb that lacks projections, except for the embolus. The carapace exhibits a shallow median groove and the female abdomen shows no posterior pockets.

**New information:**

A spider survey conducted in June 2024 from the Jiangxi Qiyunshan National Nature Reserve, Jiangxi, China recorded the genus *Khorata* for the first time. Based on morphological comparison, one new species was identified and is described here. A detailed description, diagnosis, photographs and distribution map of the new species are provided.

## Introduction

The genus *Khorata* Huber, 2005, belonging to the subfamily Pholcinae C.L. Koch, 1850, is mainly distributed in China and Southeast Asia (World Spider Catalog 2024). Out of 52 species of this genus, 34 are recorded in the southern provinces of China, such as Guangxi, Hunan, Fujian, Guangdong, Guizhou, Yunnan and Hainan (Tong and Li 2008, Zhang and Zhu 2009, Yao and Li 2010, Yao and Li 2013, Wei and Xu 2014, Yao et al. 2015, Yao et al. 2019, Xu et al. 2020, Yao et al. 2021, Sheng et al. 2021, Zhang et al. 2024, World Spider Catalog 2024). Qiyunshan National Nature Reserve in Jiangxi Province is located at the intersection area of Nanling and Luoxiao Mountains, boasting a forest coverage rate of 97.6%, making it a crucial area for biodiversity conservation in China (Huang et al. 2020). Moreover, it is also located in the humid monsoon climate zone in the eastern mid-subtropics, where spiders and insects can survive in warm and humid weather. Simultaneously, the reserve complex and variable topography offers a diverse habitat for species such as spiders, fostering their adaptation and evolution (Huang et al. 2020). The aim of this work is to describe a new species of *Khorata* Huber, 2005 from Qiyunshan National Nature Reserve, Jiangxi Province, China.

## Materials and methods

Specimens were collected by handpicking and beating shrubs and were kept in 95% ethanol. After dissection, the epigyne was cleared in trypsin enzyme solution before examination and photography. Specimens were examined and, measured with a Leica MZ6 stereomicroscope. Photos were taken with a Kuy Nice CCD mounted on an Olympus BX41 and stacked with Helicon Focus software (v.3.10) (Khmelik et al. 2005). The map was created using ArcMap 10.2 and then edited using Adobe Photoshop 2021 Extended (Fig. 4). Leg measurements are given in the following order: total length (femur + patella + tibia + metatarsus + tarsus). All measurements are given in millimetres (mm). The terminology used in text and figure legends follows Zhang et al. (2024). The specimens studied are deposited in the Taxidermy Museum of Gannan Normal University, Ganzhou City, China (GNNU).

Terminology and taxonomic descriptions follow Yao et al. (2021), Lu et al. (2022) and Zhang et al. (2024). Abbreviations: **ALE** = anterior lateral eye, **AME** = anterior median eye, **PME** = posterior median eye, **L/d** = length/diameter; used in the illustrations: **aa** = anterior arch, **b** = bulb, **da** = distal apophysis, **e** = embolus, **fa** = frontal apophysis, **ma** = posteromedian apophysis, **pa** = proximo-lateral apophysis, **pp** = pore plate, **pr** = procursus.

## Taxon treatments

### Khorata qiyunshanensis

Zhou sp. nov.

DCEAB58B-094A-5954-BB63-097C33D107F4

89F600D1-5D90-4692-A3EA-2A4FE95434E3

#### Materials

- **Type status:** Holotype. **Occurrence:** recordedBy: Zhou Guchun; individualCount: 1; sex: male; lifeStage: adult; occurrenceID: B957B1D7-21CE-5E2B-B862-EF7D13799596; **Taxon:** kingdom: Animalia; phylum: Arthropoda; class:  Arachnida; order: Araneae; family: Pholcidae; genus: Khorata; **Location:** country: China; stateProvince: Jiangxi; county: Chongyi; locality: Sishun Township, Qiyunshan National Nature Reserve, Xiangluba; verbatimElevation: 344; verbatimLatitude: 25°47′52.4″N; verbatimLongitude: 114°5′24.2″E; **Event:** samplingProtocol: by hand; year: 2024; month: June; day: 22; **Record Level:** institutionCode: JXQYS-24-43-01
- **Type status:** Paratype. **Occurrence:** recordedBy: Zhou Guchun; individualCount: 5; sex: 1 male, 4 females; lifeStage: adult; occurrenceID: 901112B9-04D7-526A-9DEA-31BD0FAA76E9; **Taxon:** kingdom: Animalia; phylum: Arthropoda; class:  Arachnida; order: Araneae; family: Pholcidae; genus: Khorata; **Location:** country: China; stateProvince: Jiangxi; county: Chongyi; locality: Sishun Township, Qiyunshan National Nature Reserve, Xiangluba; verbatimElevation: 344; verbatimLatitude: 25°47′52.4″N; verbatimLongitude: 114°5′24.2″E; **Event:** samplingProtocol: by hand; year: 2024; month: June; day: 22; **Record Level:** institutionCode: JXQYS-24-43-02-06
- **Type status:** Paratype. **Occurrence:** recordedBy: Zhou Guchun; individualCount: 1; sex: 1 females; lifeStage: adult; occurrenceID: F78B8BD2-0A51-5712-8C2A-F9FBAF0562C5; **Taxon:** kingdom: Animalia; phylum: Arthropoda; class:  Arachnida; order: Araneae; family: Pholcidae; genus: Khorata; **Location:** country: China; stateProvince: Jiangxi; county: Chongyi; locality: Sishun Township, Qiyunshan National Nature Reserve, Xiangluba; verbatimElevation: 344; verbatimLatitude: 25°47′52.4″N; verbatimLongitude: 114°5′24.2″E; **Event:** samplingProtocol: by hand; year: 2024; month: October; day: 1; **Record Level:** institutionCode: JXQYS-24-82-01

#### Description

**Male** (holotype, JXQYS-24-43-01): Total length 2.23 (2.46 with clypeus), carapace 0.92 long, 0.93 wide, opisthosoma 1.31 long, 0.97 wide. Leg I: 20.59 (5.18, 0.42, 5.16, 7.47, 2.36), leg II: 13.97 (3.95, 0.42, 3.39, 4.79, 1.42), leg III 10.59 (3.11, 0.37, 2.58, 3.58, 0.95), leg IV: 13.02 (3.74, 0.42, 3.23, 4.74, 0.89); tibia I L/d: 49. Eye interdistances and diameters: PME–PME 0.13, PME 0.12, PME–ALE 0.04, AME absent. Sternum width/length: 0.67/0.62. Habitus as in Fig. 2E and F. Both sides of carapace greyish-brown, the middle sides pale white; 6 white eyes, black around the eyes; sternum black with long black fine hairs. Legs brownish, but pale white on distal parts of femora and tibiae. Retrolateral trichobothrium of tibia I at 27% proximally; legs with short vertical setae on tibiae, metatarsi and tarsi, without spines or curved setae; tarsus I with 60 distinct pseudosegments. Chelicerae with pair of proximo-lateral apophyses (pa in Fig. 2C and D), pair of distal apophyses (da in Fig. 2C and D) on front-lateral surface middle, pair of strong frontal apophyses (cattle-horn shape, Fig. 2C and D), inward bending hooked frontal apophyses (fa in Fig. 2C and D; medial lower half and distance between tips: 0.05). Palp as in Fig. 3A and B; trochanter with width retrolateral apophysis (as long as wide, arrow 1 in Fig. 3B) and trochanter with dorsal apophysis (small, arrow 2 in Fig. 3B); femur with retrolateral apophysis (semi-circular ring, arrow 3 in Fig. 3B); patella large; procursus proximal slightly curved, odontoid protuberance on the lateral distally bearing scales and three small angular apophyses (arrows 1, 2 and 3 in Fig. 3C); bulb oval shape and fawn, embolus curved long strip and back end bent downwards and becomes blunt tip, its length equal to bulb.

**Figure 1.**
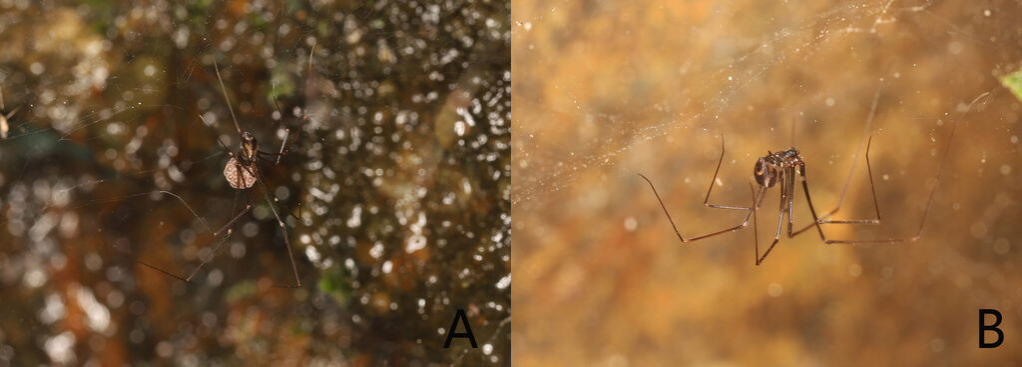
Living photos of *Khorataqiyunshanensis* Zhou, sp. nov.: **A, B** Female.

**Figure 2.**
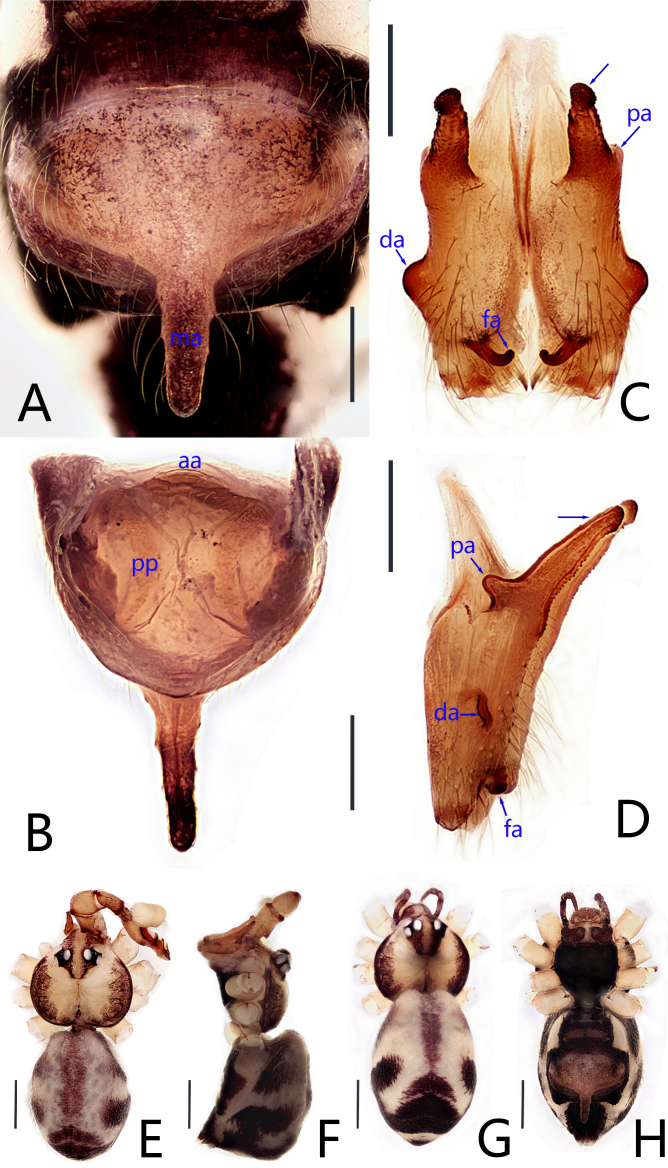
*Khorataqiyunshanensis* Zhou, sp. nov., holotype male (C–F) and paratype female (A, B, G, H). **A** epigyne, ventral view; **B** vulva, dorsal view; **C, D** chelicerae (C frontal view, D lateral view; arrows point at strong frontal apophyses); **E–H** habitus (E, G dorsal view, F lateral view, H ventral view). Abbreviations: aa = anterior arch, da = distal apophysis, fa = frontal apophysis, ma = posteromedian apophysis, pa = proximo-lateral apophysis, pp = pore plate. Scale bars: 0.20 (A–D); 0.50 (E–H).

**Figure 3.**
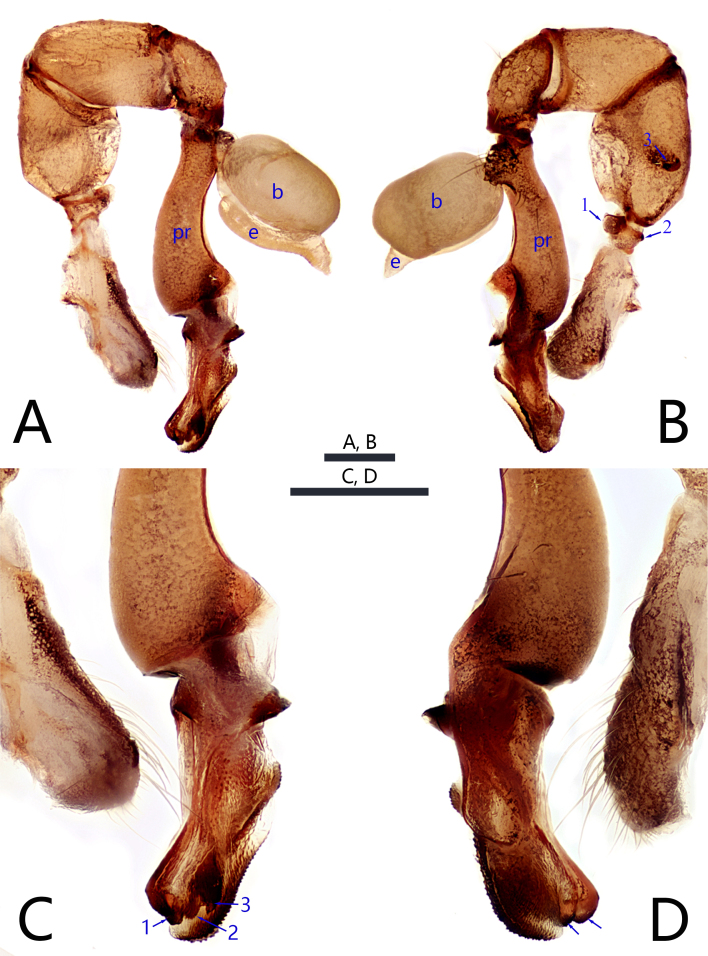
*Khorataqiyunshanensis* Zhou, sp. nov., holotype male. **A, B** palp (**A** prolateral view, **B** retrolateral view, arrow 1 points at trochanteral retrolateral apophysis, arrow 2 points at trochanteral ventral apophysis, arrow 3 points at femoral retrolateral apophysis); **C, D** distal part of procursus (**C** prolateral view, arrows 1, 2, 3 point at angular apophyses, **D** retrolateral view, arrows at angular apophyses). Abbreviations: b = bulb, e = embolus, pr = procursus. Scale bars: 0.20 (A-D).

**Female** (paratype, JXQYS-24-43-03): Similar to male, habitus as in Fig. 1A and B and Fig. 2G and H. Total length 2.35 (2.44 with clypeus), carapace 0.83 long, 0.93 wide, opisthosoma 1.52 long, 1.07 wide. Leg I: 18.32 (4.58, 0.37, 4.58, 6.37, 2.42), leg II: 11.80 (3.11, 0.32, 2.84, 4.11, 1.42), leg III 11.25 (3.05, 0.37, 2.68, 3.89, 1.26), leg IV: 12.05 (3.63, 0.37, 3.05, 4.16, 0.84); tibia I L/d: 43.5. Eye interdistances and diameters: PME–PME 0.12, PME 0.11, PME–ALE 0.02, AME absent. Sternum width/length: 0.57/0.64. Epigyne (Fig. 3A) brownish and posteromedian apophysis patent defect, outer edge black long hairs, without pockets; posteromedian apophysis (ma in Fig. 2A) long rod-shaped, its length about equal to the length of epigyne. Vulva with relief anterior arch (aa in Fig. 2B) and concave on both sides of the middle and posteriorly pointed pore plates (semi-arc, pp in Fig. 2B).

##### Variation

Tibia I in the male paratype (JXQYS-24-43-02): 3.74. Tibia I in another female paratype (JXQYS-24-43-04, 05, 06; JXQYS-24-82-01): 4.68, 4.69, 4.47; 4.71.

#### Diagnosis

**Figure 4.**
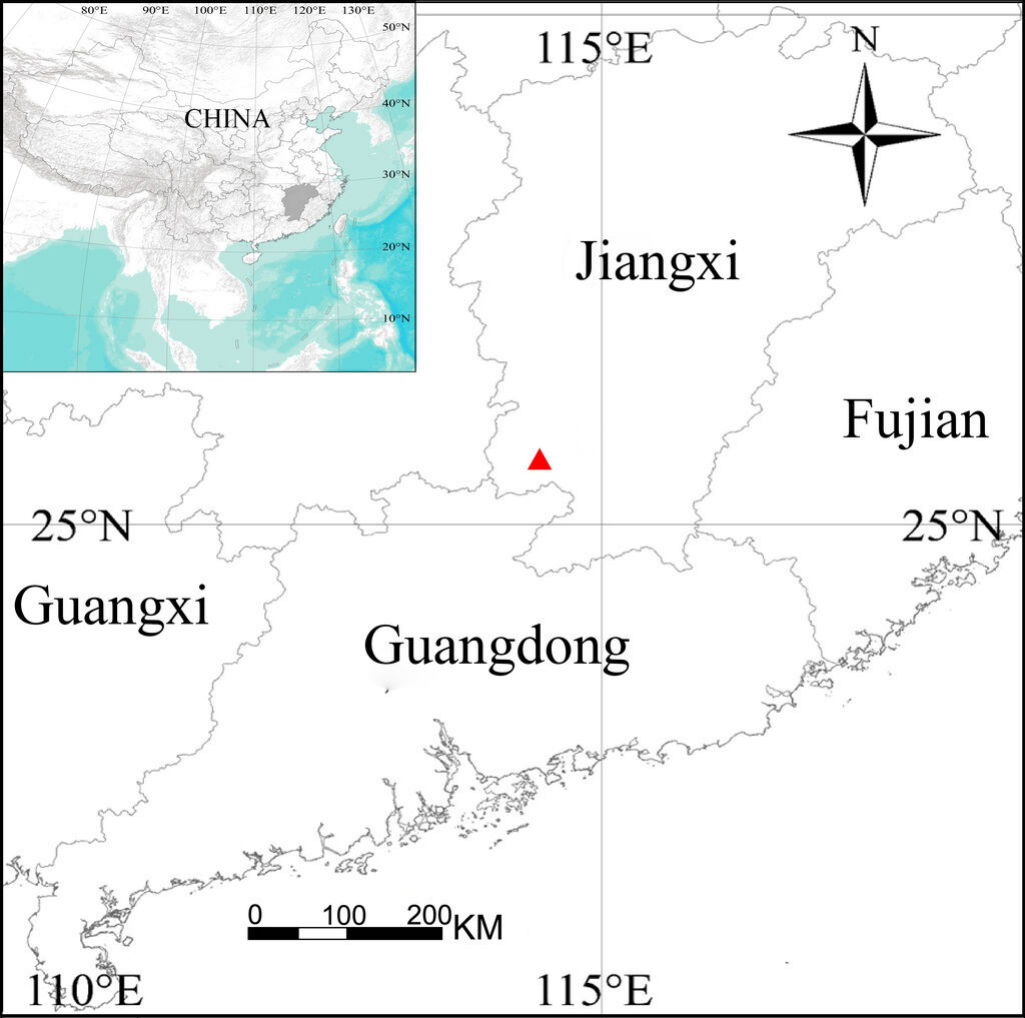
Distribution records of *Khorataqiyunshanensis* Zhou, sp. nov.

This species can be easily distinguished from all known congeners by combination of the following characters: (1) bulb oval shape and fawn, embolus length equal to bulb (Fig. 3A); (2) procursus proximal slightly curved, odontoid protuberance on the lateral distally bearing scales and three small angular apophyses (Fig. 3C); (3) chelicerae with pair of proximo-lateral apophyses (Fig. 2C and D), pair of distal apophyses (Fig. 2C and D) on front-lateral surface middle, pair of strong frontal apophyses (Fig. 2C and D), inward bending hooked frontal apophyses (Fig. 2C and D); (4) posteromedian apophysis long rod-shaped, its length about equal to the length of epigyne (Fig. 2A); (5) vulva with relief anterior arch (Fig. 2B) and concave on both sides of the middle and posteriorly pointed pore plates (Fig. 2B).

#### Etymology

The specific name refers to the type locality, adjective.

#### Distribution

China (Jiangxi, type locality; Fig. 4).

#### Biology

The species was found in the twilight zone of an artificially excavated unnamed cave or between the cliffs of Xiangluba (netting between stones).

## Supplementary Material

XML Treatment for Khorata qiyunshanensis
